# Pediatric Sedation in Dutch Dental Clinics: The Influence of Guideline Modifications on Adverse Events

**DOI:** 10.3390/dj12030066

**Published:** 2024-03-05

**Authors:** Jonah M. Hill, Daphne Y. S. Vogel, Bea Spek, Catharine J. de Jong, Janneke B. Krikken, Jaap S. J. Veerkamp

**Affiliations:** 1 Faculty of Health, Amsterdam University of Applied Sciences, Tafelbergweg 51, 1105 DB Amsterdam, The Netherlands; d.y.s.vogel@hva.nl; 2 Department of Clinical Epidemiology and Data Science, Amsterdam Universitair Medische Centra (UMC), Meibergdreef 9, 1105 AZ Amsterdam, The Netherlands; b.spek@amsterdamumc.nl; 3 Kindertand, Pediatric Dental Practice, Milletstraat 28, 1077 ZE Amsterdam, The Netherlands; cath.de.jong@xs4all.nl (C.J.d.J.); veerkampj@gmail.com (J.S.J.V.); 4 Snoet Kindermondzorgcentrum, Pediatric Dental Practice, Jan Evertsenstraat 719, 1061 XZ Amsterdam, The Netherlands

**Keywords:** dental anesthesia, pediatric dentistry, evidence-based practice

## Abstract

*Background*: Dental fear and uncooperative behavior can hinder dental treatment quality. Pediatric Procedural Sedation and Analgesia (PPSA) is used to facilitate treatment when the coping capacity is exceeded. Out-of-hospital PPSA has been associated with more adverse outcomes compared to when it is used in hospital-based settings. The updated Dutch PPSA guidelines have increased costs and raised concerns about the accessibility of specialized high-quality dental care for children in the Netherlands. This study aimed to investigate the impact of the updated 2017 guidelines on the occurrence rate of adverse events during PPSA in twelve Dutch dental clinics. *Methods*: The data of 25,872 children who were treated at twelve dental clinics between 1997 and 2019 were analyzed. A logistic two-level mixed-effects model was used to estimate the updated guidelines’ impacts on adverse events. *Results*: The OR of the occurrence rate of an adverse event adjusted for age, weight, and duration of treatment was 0.75 (95% CI 0.64–0.89) after the implementation of the updated guidelines. This outcome was significant with *p* = 0.001, indicating a protective effect. *Conclusions*: Our findings demonstrate that there was a significant reduction in adverse events after the implementation of the updated guideline and highlight the importance of adhering to evidence-based practices in out-of-hospital dental clinics.

## 1. Introduction

A quarter of the Dutch population is afraid of going to the dentist, and the prevalence of an excessive fear of the dentist in this population is 3.7% [1]. Dental fear and uncooperative behavior during dental treatment can make treatment difficult and reduces its quality.

Preventive dental care is paramount to prevent children from needing dental procedures in the first place [2]. Before children with dental fear and uncooperative behavior are eligible for more invasive techniques to make dental treatment possible, such as Pediatric Procedural Sedation and Analgesia (PPSA) or general anesthesia [3,4,5], less invasive practices should be attempted. A wide range of practices and strategies have been developed and should be considered to reduce pain and anxiety [6,7]. However, when less invasive practices do not work and dental fear and uncooperative behavior make dental treatments impossible, or when the treatment plan exceeds the coping capacity of children, PPSA can be used to make treatment possible [2,4,5]. PPSA has three main goals: preventing or relieving pain and anxiety, facilitating the procedure, and promoting patient safety [4,5]. Although rare, PPSA has not been without adverse events (AE) and mortality in the past [8,9,10].

The incidence of severe adverse events during in-hospital pediatric sedations is estimated to be 1/10,000 [11]. This estimate differs greatly compared to that of out-of-hospital pediatric sedations. In 2000, Coté published an article on “adverse sedation events in pediatrics”. The conclusion Coté made was that “adverse outcomes (permanent neurologic injury or death) occurred more frequently in a nonhospital-based facility, whereas successful outcomes (prolonged hospitalization or no harm) occurred more frequently in a hospital-based setting” [11,12].

In a 2006 review, Cravero J. et al. described several adverse events during PPSA outside of the operating room. Airway obstructions were reported in 93.2 of 10,000 sedations, allergic reactions were reported in 3.0 of 10,000 sedations, and an oxygen saturation of less than 90% was reported in 154.4 of 10,000 sedations [10]. In the dissertation of Leroy P. entitled “Improving Procedural Sedation and/or Analgesia in Children”, a concerning issue was highlighted. Despite the presence of well-established safety guidelines, three severe incidents occurred during PPSA in children. Tragically, two of these incidents resulted in fatalities, while one child suffered permanent damage. Leroy P. also noted that these events were not isolated incidents, but rather indicative of a more widespread problem involving non-compliance with established safety guidelines [13]. This underscores the critical need for the development of guidelines specifically tailored for PPSA in settings outside of traditional operating rooms.

Patient safety and the awareness of adverse events have steadily increased in Europe, leading to a number of patient safety initiatives [14,15] like “the safety management system” that ran from 2008 to 2012 in the Netherlands [16]. Evidence-based best practices and patient safety initiatives led to improvements in the PPSA guidelines and practices [11,17,18,19] and resulted in a decline in anesthesia-related mortalities [20]. The most recent PPSA guidelines published in 2017 recommended two anesthesiologists and a ventilator to be present during out-of-hospital PPSA [21]. Pre-2017 guidelines recommended having one anesthesiologist and did not mention the necessity of having a ventilator present. This modification subsequently led to higher costs of out-of-hospital PPSA and raised the concern that specialized and high-quality healthcare would become less accessible [22].

To date, we are not sure whether this costly change in procedure has any effects on adverse events. To address this uncertainty, this natural experiment study aims to estimate the influence of the updated guidelines on the occurrence of adverse events during PPSA as recorded in the anesthesia complication databases of twelve participating Dutch dental clinics. The data were obtained from children between 2 and 18 years of age and adjusted for age, body weight, and duration of treatment.

## 2. Methods

### 2.1. Setting and Participants

The data used in this study were collected between 1997 and 2019 from 12 dental clinics performing PPSA outside the hospital setting and were retrospectively analyzed. The dataset contained data from a total of 25,872 children. This study was approved by the pediatric dental group in pedodontology and anesthesiology (Kindertand-groep pedodontologie en anesthesiologie) for the use of a randomized anesthesia complication database. This study was designed as a natural experiment study because the circumstances surrounding the implementation of the 2017 guidelines were beyond our control [23].

All children referred to these specialized dental clinics were screened in the dental clinics by an anesthesiologist and a dentist. The ASA classification system (American Society of Anesthesiologists) [24] was used to assess pre-anesthesia medical co-morbidities [20]. Children ranging from 2 to 18 years with the ASA classifications I and II were eligible for PPSA. Children under two and over eighteen years, or with ASA classifications of III and upwards, were referred to a general hospital for sedation and were not included in this study.

### 2.2. Procedure

Children were referred by their own dental practitioners, school dentists, or pediatricians to one of the specialized dental clinics for Propofol sedation. Before the guidelines were updated, the designated team for dental treatment and Propofol sedation consisted of a pediatric dentist, an anesthesiologist, a designated dental assistant, a nurse anesthetist, and a designated assistant in the recovery ward. After the guidelines were updated, a second anesthesiologist was added.

In all dental clinics, a uniform protocol was adhered to for administering Propofol sedation. This protocol remained consistent even following the update of the clinical guidelines. According to this protocol, patients weighing less than 20 kg received a dosage of 5 mg/kg of Propofol, those in the weight range of 20–30 kg were administered 4 mg/kg of Propofol, individuals weighing 30–40 kg received 4 mg/kg of Propofol, and those exceeding 40 kg were given 3 mg/kg of Propofol. Subsequently, the maintenance dose was initiated at 20 mg/kg/h and was subsequently reduced to 10 mg/kg/h after the initial hour of administration.

The protocol used for the selection of the children did not change after the guidelines were updated. All dental clinics within this study followed the guidelines set by the NVA and, if needed, included children based on clinical insights of the anesthesiologist and dentist. After the dental treatment plan was completed, the children were referred back to their own dental clinics.

### 2.3. Ethical Considerations

The database used for this retrospective study consists of data from 25,872 children collected between 1997 and 2019. Given the large number of children and the time in which the data were collected, it is “reasonably impossible to ask for permission” for the use of the data [25].

The children who were treated in the dental clinics in this study are considered vulnerable. The children may have been neglected by their parents or legal guardians, leading to the necessity of extensive dental treatment. For this reason, “a selective response is expected to preclude reliable outcomes” [25]. Written permission was given by the pediatric dental group in pedodontology and anesthesiology (Kindertand-groep pedodontologie en anesthesiologie) for the use of the database. Individual subjects could not withdraw from this study.

### 2.4. Data Collection

In the practice of PPSA in the Netherlands, it is a legal requirement to record any adverse events [26]. In this study, adverse events were tracked using a specialized anesthesia complication database, which included specific predetermined variables such as the clinic code, treatment date, patient’s age, treatment duration, the total amount of Propofol administered, and the patient’s weight, height, and Body Mass Index (BMI). These adverse events encompassed scenarios, such as more than three failed intravenous attempts, issues with the laryngeal mask, oxygen desaturation below 90%, a subcutaneous administration of Propofol, allergic reactions, and various other miscellaneous adverse events.

### 2.5. Data Analysis

The data were analyzed with RStudio version 2022.12.0 + 353. Descriptive statistics were used to summarize the baseline characteristics of the study population. The following packages were used in R studio: “tableone” for descriptive statistics, “lme4” for the construction of the model, and “DHARMa” to check the model’s assumptions. Table 1 and Table 2 provide an overview of the baseline variables per clinic. The data are expressed as the mean (SD) for continuous variables with a normal distribution and as the median (IQR) for continuous variables without normal distribution. Categorical variables are expressed in percentages.

The model was pre-defined. The selection of variables in the study was determined based on expert judgment, clinical experience, and the existing literature rather than through statistical methods. Not all clinics implemented the updated guidelines in the same year. The implementation of the updated guidelines in each clinic was recorded and dichotomized. Since a cluster effect within the Dutch dental clinics was expected [27], a logistic two-level mixed-effects model with a random intercept and fixed slope was chosen to estimate the influence of the updated pediatric sedation guidelines on the occurrence of adverse events, and a binominal distribution was used for adverse events (dependent variable). An estimate of nine clinics was calculated as a fixed effect and reported in OR with a 95% CI. The random effects were reported as variances and standard deviations of the odds and the probability of an adverse event per clinic. For the regression coefficient b value, the Wald test was used to indicate the significance of the association with the outcome. The *p* value for statistical significance was set at *p* < 0.05. Log-link function was used to interpret the odds of adverse events and to define the odds ratio for the effect of the guidelines, adjusted for the weight of the child and duration of treatment.

### 2.6. Missing Data

Multiple imputation was applied if >5% of the variables were missing. A correlation matrix and a correlation plot were made to assess whether patterns of missing data could be attributed to missing completely at random (MCAR) or missing at random (MAR). The correlation matrix was used to analyze whether there were pairs of variables with missing data. Additionally, a matrix plot was used to determine whether there were relations between the missing variables. The determination was made by assessing whether the missing data exist because of the variable itself to rule out missing not at random (MNAR). A complete case analyzation would be made with variables that are MNAR.

## 3. Results

The total anesthesia complication dataset contained data from 25,872 children from 12 dental clinics collected between 1997 and 2019. Three clinics did not work with or did not exclusively work with the updated guidelines from 2017. A contrast is needed before and after the implementation of the updated guidelines to estimate the influence of the updated pediatric sedation guidelines on adverse events. As a result, these clinics were not included in this study. The total number of children analyzed in the remaining nine clinics was 21,759.

Of the 21,759 observations, information about the treatment time was missing 39 times (0.18%), and information about weight was missing 18 times (0.08%), with both values being less than 5 percent. Information about height and BMI was missing 9027 times (41.5%). Multiple imputations were considered for the variables, but as expected, a relationship was found in the matrix plot between the length of missing variables and BMI in a number of dental clinics. These were not randomly distributed among the dental clinics. As a result, no multiple imputations were applied for these variables. A complete case analyzation was made with the variables of treatment time, weight, and age.

In the logistic two-level mixed-effects model, when adjusting for age, weight, and treatment duration, the odds ratio (OR) for the occurrence of adverse events was 0.75 (95% CI 0.64–0.89) following the implementation of the updated guidelines compared to the period before its implementation. This result was statistically significant with a *p*-value of 0.001. Furthermore, among the nine dental clinics under consideration, a variance of 0.05 and a standard deviation of 0.23 were observed in the occurrence of adverse events. On average, the odds of experiencing an adverse event per clinic were 0.04, corresponding to a 3.81% probability (as indicated in Table 3).

## 4. Discussion

This natural experiment aimed to assess the impact of the updated guidelines on the occurrence of adverse events during the administration of PPSA within the context of twelve participating Dutch dental clinics using data from an anesthesia complication database. The findings revealed a significant protective effect associated with the implementation of the updated guidelines, resulting in a statistically significant reduction in the occurrence of adverse events. Specifically, the odds of experiencing adverse events decreased by 25% (OR 0.75) following the update of the guidelines in comparison to the period preceding the update (Table 3). This demonstrated a significant decline in the occurrence of adverse events following the updated guidelines’ implementation. Furthermore, it is worth noting that substantial variability in the occurrence of adverse events was observed among the nine clinics, indicating the potential presence of a cluster effect within these clinical settings (Table 3). This underscores the importance of considering clinic-specific factors when evaluating the impact of clinical guidelines. 

Bainbridge et al. described in their systematic review that anesthetic-related mortalities have steadily declined over the past 50 years in developed countries, with a higher rate of improvement being found in these regions. They emphasized the importance of evidence-based best practices in reducing anesthetic-related mortalities in developing countries [20]. In this study, a steady accumulation of evidence-based guidelines in developed countries was found as was observed in the implementation of the updated PPSA guidelines of 2017 in Dutch dental clinics. The protective influence of the updated guidelines may be a part of the continued development of evidence-based guidelines and safety initiatives [20]. 

In this study, a natural experiment design was adopted due to the circumstances of the revised 2017 guidelines’ implementation, which were outside of this study’s control [23], precluding the possibility of a prospective study design. The random assignment of participants and clinics to intervention or control groups was not feasible. The implementation of Pediatric Procedural Sedation and Analgesia (PPSA) commenced variably across dental clinics during the years of 1997 to 2019. Additionally, the application of the revised 2017 guidelines occurred at disparate times for each clinic, ranging from 2016 to 2018. Consequently, this led to differential exposure to evolving evidence-based practices among the clinics. A strength of this study is its ability to use a natural experiment study design. A natural experiment can be seen as a robust alternative to an RCT [23]. Natural experiments use real-life systems rather than using a system that is designed or modified for the purpose of research, such as in an RCT [28], creating greater external validity. A natural experiment study design has greater generalizability, which contributes to our insight in “real-world” occurrences of out-of-hospital-PPSA-related adverse events in Dutch dental clinics. The use of a large dataset also allows for adequate statistical power. In the final model, nine of the twelve clinics were analyzed, with a total of 21,815 children and 1203 adverse events, allowing adequate statistical power to be obtained. 

The lack of control over the evolution of evidence-based practices and the implementation of safety initiatives from 1997 to 2019 across individual dental practices may have introduced limitations to the internal validity of this study. Variability in exposure to these factors could result in distinct impacts on the occurrence of adverse events in each clinic. In anticipation of a cluster effect within the Dutch dental clinics [27], a two-level logistic mixed-effects model was employed, positioning the dental clinics at the first level and the occurrence of adverse events at the second level.

When evaluating the findings of this study, the presence of a potential learning effect needs to be acknowledged. Dental clinics that joined the study at a later stage had the advantage of tapping into the collective experience, enhanced clinical expertise, and assimilated knowledge and best practices gained from clinics previously participating in pediatric sedation. This accrued knowledge might have played a role in reducing adverse events independently of the impact of the updated guidelines. Consequently, this factor has the potential to introduce a confounding element into the established association between the implementation of the guidelines and the observed reduction in adverse events. Despite this potential confounder, it is important to underscore the robustness of this study. It provides valuable insights into the impact of the updated guidelines on adverse event rates in dental clinics, serving as a significant contribution to the field. The consideration of the learning effect enhances this study’s credibility as it demonstrates a comprehensive approach to account for potential sources of influence on the observed outcomes.

From the anesthesia complication dataset, which predominantly encompasses adverse events defined in less precise terms, this can be considered as a limitation. In contrast, the Pediatric Sedation Research Consortium [10,29,30] employs meticulously defined criteria for adverse events. Notably, the only adverse event sharing an identical definition with the consortium’s criteria is desaturation below 90%. Conversely, adverse events, such as those involving more than three intravenous attempts, complications with the laryngeal mask, subcutaneous Propofol administration, allergic reactions, and miscellaneous adverse events, are less congruent with the definitions employed by the consortium [30]. This divergence in adverse event definitions poses a challenge when attempting to draw direct comparisons with the outcomes reported by the consortium. To enhance the quality of future research, a potential solution lies in standardizing the definitions of adverse events within a forthcoming complication database. Such standardization would facilitate more robust and meaningful comparisons in future studies, solidifying this study’s contribution to the field.

## 5. Conclusions

After the introduction of the 2017 guidelines on PPSA in out-of-hospital Dutch dental clinics, a significant reduction of 25% (OR 0.75) in the occurrence of adverse events was observed. At the beginning of this study, there were uncertainties regarding the possible impacts of the costly procedural changes on adverse events. This study’s outcomes, however, reveal a significant reduction in adverse events after the implementation of the updated guidelines and highlight the importance of adhering to evidence-based guidelines in improving patient safety and the quality of care provided during pediatric procedural sedation and analgesia in out-of-hospital dental clinics. This study underlines the necessity of considering the complex underlying mechanisms that play roles in the development and implementation of evidence-based practices and safety initiatives regarding the occurrence of adverse events.

## Figures and Tables

**Table 1:** Baseline variables of analyzed dental clinics before release of 2017 guidelines.

Clinic Number	Clinic 2	Clinic 3	Clinic 4	Clinic 5	Clinic 6	Clinic 7	Clinic 8	Clinic 9	Clinic 11	Total
N children	7133	3256	1219	816	2272	498	384	1365	199	17,142
Age (median, IQR)	4 (3–5)	4 (3–6)	4 (4–6)	4 (3–5)	5 (4–6)	5 (4–6)	5 (4–6)	5 (4–7)	4 (4–6)	4 (3–6)
Weight in kg (median, IQR)	18 (16–21)	18 (16–23)	18 (16–23)	18 (16–21)	20 (17–24)	19 (16–23)	19 (17–23)	20 (17–26)	20 (17–24)	18 (16–22)
Length in cm (median, IQR)	110 (103–120)	113 (105–124)	112 (104–126)	108 (102–116)	114 (106–124)	113 (105–122)	111 (104–122)	117 (107–129)	112 (105–120)	112 (104–123)
BMI (median, IQR)	15 (14–17)	15 (14–16)	15 (14–16)	16 (14–17)	15 (14–17)	16 (14–17)	16 (15–17)	15 (14–17)	16 (15–17)	15 (14–17)
Propofol in mg (median, IQR) *	490 (392–610)	485 (387–603)	529 (420–670)	510 (420–620)	550 (450–678)	481 (370–630)	492 (422–580)	504 (400–641)	570 (464–700)	500 (400–626)
Treatment time (median, IQR) **	60 (47–73)	55 (42–65)	67 (50–90)	75 (60–90)	60 (50–70)	70 (60–86)	50 (45–60)	60 (50–75)	65 (60–78)	60 (50–75)
Recorded adverse events (%)	496 (7.0)	172 (5.3)	92 (7.5)	29 (3.6)	108 (4.8)	31 (6.2)	25 (6.5)	51 (3.7)	9 (4.5)	1013 (5.9)

* Mean Propofol dosage per session in milligrams. ** Mean duration of PPSA in minutes.

**Table 2:** Baseline variables of analyzed dental clinics after release of 2017 guidelines.

Clinic Number	Clinic 2	Clinic 3	Clinic 4	Clinic 5	Clinic 6	Clinic 7	Clinic 8	Clinic 9	Clinic 11	Total
N children	935	471	330	163	877	156	185	833	723	4673
Age (median, IQR)	5 (4–6)	5 (4–7)	6 (4–9)	4 (3–6)	5 (4–6)	5 (4–6)	5 (4–6)	6 (4–7)	4 (4–6)	5 (4–7)
Weight in Kg (median, IQR)	20 (17–23)	20 (17–26)	21 (17–30)	19 (16–23)	20 (17–25)	21 (18–25)	21 (17–25)	21 (18–27)	20 (17–24)	20 (17–25)
Length in cm (median, IQR)	110 (104–120)	114 (105–128)	119 (106–137)	110 (102–122)	114 (106–125)	119 (110–128)	116 (106–124)	119 (109–130)	110 (103–120)	114 (105–125)
BMI (Median, IQR)	16 (15–17)	16 (15–17)	15 (14–17)	15 (14–17)	16 (15–17)	16 (14–17)	16 (15–17)	15 (14–16)	16 (15–18)	16 (15–17)
Propofol in mg (median, IQR) *	510 (424–630)	505 (419–626)	500 (400–658)	510 (434–600)	475 (386–580)	510 (420–617)	532 (456–644)	520 (420–660)	520 (421–630)	505 (411–625)
Treatment time (median, IQR) **	65 (55–75)	55 (45–65)	60 (45–75)	70 (60–85)	60 (50–65)	70 (55–80)	55 (50–65)	65 (55–80)	60 (45–70)	60 (50–75)
Recorded adverse events (%)	49 (5.2)	23 (4.9)	19 (5.8)	4 (2.5)	15 (1.7)	2 (1.3)	10 (5.4)	37 (4.4)	31 (4.3)	190 (4.1)

* Mean Propofol dosage per session in milligrams. ** Mean duration of PPSA in minutes.

**Table 3:** Odds of adverse event before [1] and after [3] implementation of updated guidelines. Probability of adverse event before [2] and after [6] implementation of updated guidelines. Odds of adverse event before and after implementation of updated guidelines combined [7]. Probability of adverse event before and after implementation of updated guidelines combined [4].

Adverse Events per Clinic *	Odds [1]	Percentage [2]	Odds [3]	Percentages [6]	Odds [7]	Percentage [4]
Clinic no. 2	0.05	4.99%	0.04	3.74%	0.05	4.90%
Clinic no. 3	0.04	3.91%	0.03	2.93%	0.04	3.84%
Clinic no. 4	0.05	4.88%	0.04	3.66%	0.05	4.79%
Clinic no. 5	0.03	2.65%	0.02	1.99%	0.03	2.61%
Clinic no. 6	0.03	3.23%	0.02	2.42%	0.03	3.18%
Clinic no. 7	0.04	3.77%	0.03	2.83%	0.04	3.71%
Clinic no. 8	0.05	4.68%	0.04	3.51%	0.05	4.60%
Clinic no. 9	0.04	3.45%	0.03	2.59%	0.04	3.39%
Clinic no. 11	0.04	4.01%	0.03	3.01%	0.04	3.96%
Overall adverse events in all clinics *	0.04	3.95%	0.03	2.96%	0.04	3.81%

* Adverse events adjusted for treatment time, age, and weight.

## Data Availability

Restrictions apply to the availability of these data. The data were obtained from Kindertand, Pediatric Dental Practice, Milletstraat 28, 1077 ZE, Amsterdam, the Netherlands and are available from Catherine de Jong (cath.de.jong@xs4all.nl) with the permission of Kindertand, Pediatric Dental Practice.
